# Recent Progress of Magnetically Actuated DNA Micro/Nanorobots

**DOI:** 10.34133/2022/9758460

**Published:** 2022-02-07

**Authors:** Fengyu Liu, Xiaoming Liu, Qiang Huang, Tatsuo Arai

**Affiliations:** ^1^Key Laboratory of Biomimetic Robots and Systems, Ministry of Education, State Key Laboratory of Intelligent Control and Decision of Complex System, Beijing Advanced Innovation Center for Intelligent Robots and Systems and School of Mechatronical Engineering, Beijing Institute of Technology, Beijing 100081, China; ^2^Center for Neuroscience and Biomedical Engineering, The University of Electro-Communications, Tokyo 182-8585, Japan

## Abstract

In the past few decades, the field of DNA origami-based micro/nanotechnology has developed dramatically and spawned attention increasingly, as its high integrality, rigid structure, and excellent resistance ability to enzyme digestion. Many two-dimensional and three-dimensional DNA nanostructures coordinated with optical, chemical, or magnetic triggers have been designed and assembled, extensively used as versatile templates for molecular robots, nanosensors, and intracellular drug delivery. The magnetic field has been widely regarded as an ideal driving and operating system for micro/nanomaterials, as it does not require high-intensity lasers like light control, nor does it need to change the chemical composition similar to chemical activation. Herein, we review the recent achievements in the induction and actuation of DNA origami-based nanodevices that respond to magnetic fields. These magnetic actuation-based DNA nanodevices were regularly combined with magnetic beads or gold nanoparticles and applied to generate single-stranded scaffolds, assemble various DNA nanostructures, and purify specific DNA nanostructures. Moreover, they also produced artificial magnetism or moved regularly driven by external magnetic fields to explain deeper scientific issues.

## 1. Introduction

Inspired by the Holliday junction, a four-way junction intermediate observed during recombination [1], Seeman first proposed thermodynamically stable four-way junctions with sticky ends and built double crossover and triple crossover motifs [2]. Based on these motifs, the tile structures were used to construct more complicated tile complexes to manufacture further nanoribbons and nanotubes [3, 4]. However, the design of tile-based DNA nanostructure was cumbersome, with the assembly requiring strictly balanced stoichiometry, and the structure was also limited by the length of the synthetic oligonucleotides [5]. Rothemund raised “DNA origami” in 2006 [6], a more recent method for assembling the desired structure via folding an ultralong single-stranded “scaffold” into a custom-designed shape by hybridizing with hundreds of short “staple” strands. Many researchers used programmed DNA origami to design and produce two-dimensional (D) or three-dimensional (3D) nanostructures [6–10].

The high capacity and chemical stability of DNA make it an ideal platform for data storage. This innovative technique was first proposed in 1988 [11]. A further step was investigated by Goldman and colleagues [12], they stored ASCII text, JPEG, and MP3 file formats in DNA and then shipped the DNA worldwide under standard conditions. Many researchers integrated the inherent property of DNA: data storage with the structural scaffold of DNA and then discovered more complicated applications from this high fidelity and enduring material. Fan group constructed an M13 viral scaffold into nanometer-scale self-assembled braille-like patterns, creating a key over 700 bits [13]. They stated that specific nanostructures could be used for secure communication with the help of intrinsic nanoscale addressability of DNA origami structures. Recently, Hughes and coworkers demonstrated a binary approach for nucleic acid memory with the assistance of DNA-PAINT superresolution microscopy. The invented error-correction included fountain and bilevel parity codes. Their research indicated that even some messages were missed from DNA origami; the error-correction method still worked and fully recovered these missing [14].

Moreover, aiming to expand the application and development of DNA origami technology in multiple research fields, custom-designed nanostructures were also utilized as nanotemplates for conjugating additional functional elements, such as proteins, aptamers, magnetic beads, gold nanoparticles (AuNPs), and quantum dots [15–19]. The loading of additional materials means that they can endow dynamic changes on DNA nanostructures, which could be ignited by optical, electrical, and chemical triggers. A reconfigurable DNA nanotweezer was reported by Stephanopoulos et al. The nanotweezer could be switched between a closed and open state with a brief UV exposure [20]. In 2018, Simmel group integrated a robotic arm onto a DNA-based molecular platform and demonstrated the robotic arm could be actuated with externally applied electrical fields [21]. In cancer therapy, Ding and collaborators developed a DNA tubular nanorobot that could provide specific cargo delivery in response to a molecular trigger [22]. So far, the majority of conformational changes of DNA origami structures could be realized through external stimuli. However, these outside triggers might directly stimulate the DNA nanostructures and inaccurately control the motions or rotations.

Magnetically actuated micro/nanorobots have attracted considerable research interests and have been developed rapidly in recent years because of their advantages, such as untouched control, insensitivity to biological substances, and precise positioning [23–26]. Under the dominance of the magnetic fields, many investigators employed the micro/nanomachines assembled by DNA origami frameworks to gain a series of pivotal applications, including single-molecule force spectroscopy to material property, drug delivery for cancer treatment, and high-sensitivity detection based on ELLSA [27–29]. According to various tasks, magnetic DNA micro/nanorobots can be categorized into property characterization, multitarget sorting, sensitive detection, and cargo delivery [29–32]. Corresponding to electromagnetic fields, there are traditional permanent magnetic fields and novel electromagnetic resonances generated by DNA origami-guided nanogold arrays [28, 33, 34]. Although massive critical papers have classified the many DNA micro/nanorobot applications and provided outstanding insights for further development. We still believe that a comprehensive review focusing on magnetically actuated DNA micro/nanorobots is quite helpful for further research on the design and manufacture of DNA micro/nanorobots that magnetic fields could control.

In this paper, we systematically described the detailed progress of magnetic field-guided target screening in the generation of DNA single-stranded scaffolds, the assembly of DNA nanostructures, and the high-throughput sorting of DNA nanostructures. We also categorize and analyze related studies based on biomedical applications, electromagnetic applications, and mechanical motions. Moreover, we scientifically investigate the current defects of magnetic DNA micro/nanorobot self-assemblies and the future directions of magnetic field constructions. We finally discuss and look forward to the research potential and development significance of magnetic DNA micro/nanorobots.

## 2. Generation and Purification of Magnetically Actuated DNA Structures

### 2.1. Single-Stranded Scaffold Generation

**Figure 1:**
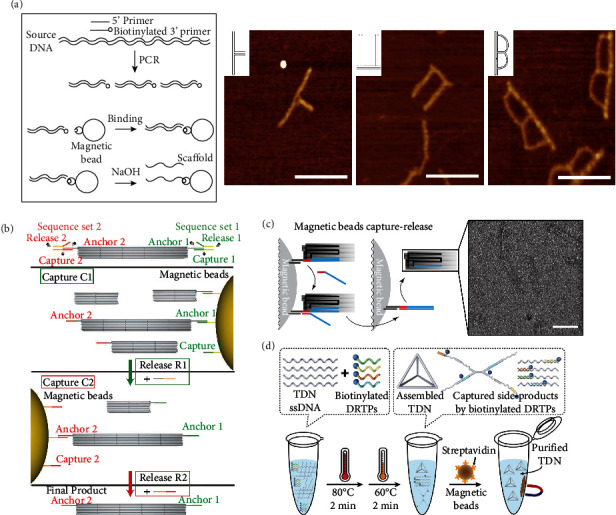
Scaffold generation, real-time folding, and targeted purification of DNA origami structure guided by magnetic beads. (a) The pathway of single-stranded scaffold generation and the representative AFM images of different DNA origami nanoletters. Scale bars: 200 nm. Reprinted with permission from Ref. [39]. Copyright 2009 American Chemical Society. (b) Route diagram of sequential pull-down procedure applied to a linear pentameric superstructure of origami tubes. Adapted from Ref. [31] with the permission under the terms of the CC-BY-NC 4.0 license. (c) After adding the invader (red-blue line), the nanostructure captured by magnetic beads was released into the recovery buffer. TEM micrograph showed the IgG-18HB purified by magnetic beads capture-release. Scale bar: 100 nm. Reprinted with permission from Ref. [43]. Copyright 2015 American Chemical Society. (d) Schematic illustration of the one-pot synthesis and purification of TDN. Adapted with permission from Ref. [48]. Copyright 2021 American Chemical Society.

DNA nanostructures can be synthesized from oligonucleotides [35] or fabricated using the origami method with the help of a long single-stranded (ss) scaffold [36, 37]. The size of the DNA nanostructures is closely associated with the length of the ssDNA. Therefore, accurately obtaining the ssDNA of various lengths is necessary for assembling DNA nanostructures with multiple sizes and shapes. Pound and coworkers developed an effective strategy to produce ssDNA by employing streptavidin-coated magnetic beads to capture specific biotinylated primers that are complementary to the original double-stranded (ds) DNA in the polymerase chain reactions, and then utilized the denaturation of sodium hydroxide [38] to purify the ssDNA from the original materials (Figure 1(a)) [39]. Specifically, 4300 ± 700 ng of dsDNA (2958 bp) combined with 800 ng of streptavidin-coated magnetic beads could yield 1100 ± 300 ng of ssDNA [39]. They also successfully synthesized thin, branched nanoletters instead of filled shapes (Figure 1(a)). They claimed that these novel DNA nanodevices could be used as circuit templates to solve potential problems such as narrow and branched wiring.

### 2.2. DNA Structure Assembly

The one-pot thermal annealing reaction is the most commonly used method for assembling DNA origami structures. High-temperature denaturation (over 80°C) can eliminate the secondary or tertiary structure [9] of ssDNA, which may accelerate the binding of staple strands. In this reaction, it is almost impossible to separate the individual elementary processes and comprehend the role of each process. Koster and colleagues successfully dissected the self-assembly process of DNA origami structures using single-molecule force spectroscopy [40] based on magnetic tweezers [41]. This assembly was divided into three main projects: the mechanical stretching of the scaffold DNA, base-pairing with staple strands, and displacement between the bound staple strands. They also proposed the parallel folding method to induce the folding of many scaffold DNA in one folding cycle and monitored their fluorescence resonance energy transfer (FRET) efficiencies. Their findings [42] prove that this novel magnetic tweezers-based assembly strategy could be carried out quickly and accurately in a parallel manner.

### 2.3. Purification of Assembled DNA Structures

Using individual DNA origami building to construct high-order superstructures is a promising pathway for creasing the structure dimensions and complexity. However, how to separate the fully assembled superstructures from the solution containing the plentiful substructures without damage or waste is still a thorny issue. As an alternative method of DNA nanostructure purification, the use of magnetic beads to extract affinity-based sample molecules from the solution has been recently established [19]. Ye and colleagues skillfully used different magnetic beads precoated with specific capture strands, and then they tried to purify the linear superstructures from the excess substructures [31]. It should be noted that the prerequisite for the successful implementation of the sequential pull-down scheme is the establishment of highly specific, orthogonal sequence sets for capture and anchor strands (Figure 1(b)). In addition, they even achieved high recovery yields in the three-sided pull-down reactions of the T-shaped superstructures.

The high programmability of DNA origami makes it possible to address some functional elements onto the nanostructures with nanometer precision. However, the realization of functionalized DNA origami nanostructures was still affected by imperfect purification steps. Shaw et al. employed the magnetic beads purification method to compare the recovery yield of DNA origami nanostructures carrying three different targets (Alexa 488 fluorophore represent small molecules; human IgG1 represent medium-sized protein; ferritin represent large protein). Experimental data confirmed that magnetic beads capture was a potential universal purification method for DNA origami nanostructures, as its purification efficiency was not significantly related to the chemical contaminants (Figure 1(c)). This method exhibited superior purity and comparable recovery yield [43] compared with other purification methods, such as ultrafiltration, gel filtration, glycerol density gradient ultracentrifugation, polyethylene glycol precipitation, and agarose gel extraction [18, 44–47]. Recently, Gao and coworkers have designed the DNA reaction termination probes (DRTPs) for the one-pot reaction of tetrahedral DNA nanostructure (TDN) synthesis [48]. Importantly, they found that the formation of TDN was dominated by simultaneous hybridization, whereas its undesirable side products were mainly caused by step-wise hybridization. Based on this crucial discovery, they optimized the reaction temperatures and introduced streptavidin-modified magnetic beads targeting biotin-labeled DRTPs into the purification steps of TDN, increasing the average yield of TDN from 81.3% to 97.4% (Figure 1(d)).

## 3. Mechanical Motions of DNA Micro/Nanorobots Coordinated with Magnetic Fields

Benefiting from the simple construction and passive infinite-bandwidth force clamps of magnetic tweezers, some researchers used them to explore DNA topoisomerase [49] and F_0_F_1_ ATPase [50]. In a typical magnetic tweezer depicted by Strick et al. [51], the micromanipulation chamber was mounted on an inverted microscope. The permanent magnets were placed on the micromanipulation chamber to provide an upward pulling force to the target magnetic beads. Moreover, the controlled rotation of the magnetic field can be realized by coupling the permanent magnets with the rotating motors under the precise control of the computer. In addition to the permanent magnet configuration, magnetic tweezers could be designed with electromagnets [52, 53]. By changing the current parameters of the electromagnets more simply and programmatically than permanent magnets, the magnetic tweezers assembled with the electromagnet dynamically controlled the motion or rotation of the target particle without any movement. However, a high current was required to generate enough force to drive the magnetic beads, and substantial heat was also generated inside the coil. Therefore, the more suitable application for electromagnet-based magnetic tweezers was that the required magnetic field was not very strong so that the magnetic tweezers could be as close to the target particles as possible to weaken the current that controls the magnetic field. Moreover, equipping the electromagnetic coil with a water-cooled device was another good choice [54].

### 3.1. Force-Calibrated Extensions

**Figure 2:**
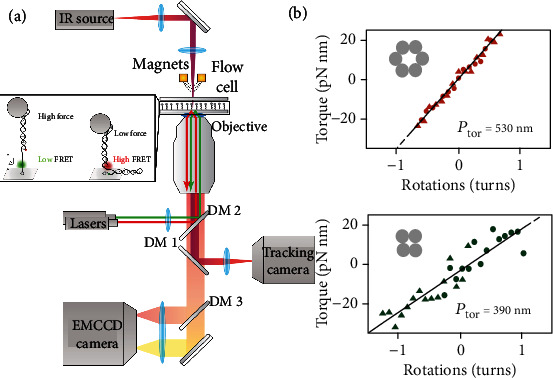
Using the permanent magnet-based magnetic tweezers to measure the force changes of DNA origami structures. (a) A complicated system combined with high-resolution magnetic tweezers and dual-color single-molecule fluorescence detection. Magnifying cartoon showed the DNA sample, consisting of a 40 bp hairpin that is flanked by a 5.9 kb double-stranded DNA spacer. Donor and acceptor fluorophores for FRET detection are shown in green and red, respectively. Concretely, at high force, the hairpin opens, while it is closed at low forces. Reprinted with permission from Ref. [30]. Copyright 2016 American Chemical Society. (b) Linear fits the data (solid black lines) provide torsional persistence lengths of 530 ± 20 nm and 390 ± 30 nm for the 6HB (d) and 4HB (e), respectively. 2.0 pN (red circles) and 3.7 pN (red triangles) referred to the 6HB; 9 pN (green circles) and 6 pN (green triangles) referred to the 4HB. Reprinted with permission from Ref. [28]. Copyright 2011 American Chemical Society.

Single-molecule force spectroscopy has emerged as an attractive tool for studying motions and forces related to biomolecules and enzyme activity [55]. The minute forces and mechanical properties of biomolecules could be measured at the single-molecule level by using the magnetic tweezer to overcome the oxygen radicals caused by the lasers [56]. However, due to the limited wavelength range for fluorescence excitation and emission, combining magnetic particle tracking and synchronous fluorescence detection was technically challenging. Kemmerich and coauthors developed a powerful instrument that simultaneously monitors DNA hairpin and Holliday junction configuration changes in two different output channels by combining high-resolution magnetic tweezers and dual-color single-molecule fluorescence detection. The robust device (Figure 2(a)) decreased the delay time of synchronization to 3 ms and enhanced the acquisition rates of the magnetic tweezer experiments at subnanometer accuracy to 2 kHz [30].

3D DNA architectures have been widely constructed and applied to various fields as rigid mechanical mediators or force sensing elements. Thus, the material properties of complex DNA architectures are urgently required to be elaborated, such as bending rigidity and torsional rigidity. Kauert et al. applied permanent magnetic tweezers to measure and analyze these properties of four-helix and six-helix bundles [28]. To quantitatively evaluate the bending rigidity of the multihelix bundles, they characterized the force-extension behavior for each investigated nanostructure, where the extension was the mean elevation of the fluctuating bead from the surface. The force-extension behavior data indicated that the bending stiffness of 4 Helix Bundle (HB) and 6HB was 15-folds and 38-folds stronger than floppy DNA duplexes. The torsional persistence lengths of 4HB and 6HB constructs were also measured. According to the linear fits of the direct measurements, the torsional rigidity of 6HB and 4HB was increased by 4.0 times and 5.5 times compared to DNA duplexes (Figure 2(b)). Finally, they concluded that the bending rigidity of multiple DNA bundles was significantly increased while the twisting rigidity was increased moderately compared to DNA duplexes. In contrast, the experimental data from these papers [35, 57] suggested that the bending rigidity of DNA multihelix bundles was consistent with the mechanical model of a single DNA duplex. In addition, their models demonstrated that interruptions of DNA duplexes by nicks at staple ends and by the Holliday-junction crossovers had only a minor influence on the mechanics of DNA origami structures.

### 3.2. Rotational Movements

**Figure 3:**
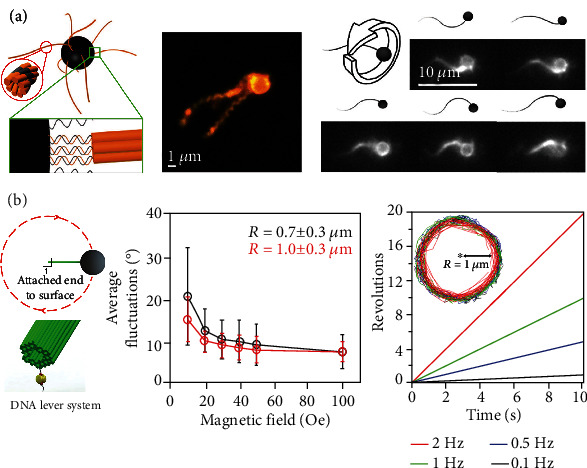
The combinations of DNA nanostructures and magnetic particles were manipulated by electromagnetic tweezers. (a) Left image: assembly drawing of DNA-flagellated magnetic bead hybrid. The red circle displayed the twisted 8-helix (tw8HT) design. The green box exhibited the biotin-streptavidin coupling between the DNA-modified magnetic beads and the DNA tile-tube. Middle image: The DNA-flagellated magnetic bead hybrid (tw8HT) was imaged in 75% glycerol to slow down thermal fluctuations, increasing the twist diameter. Right graph: schematic diagram of the tw8HT hybrid structure driven by a homogeneous magnetic field and respective fluorescence microscopy images, which rotates perpendicular to the swimming direction. Reprinted from Ref. [54] with the permission under the terms of the CC-BY license. (b) Left graph: illustration of the DNA lever system. The DNA lever was attached to the surface via biotin-streptavidin affinity. Middle chart: The average and standard deviation of the in-plane angular fluctuations across all 13 DNA levers (black traces). The red traces represent the average of the four longest DNA levers. Right chart: DNA levers were actuated at four frequencies 0.1, 0.5, 1, and 2 Hz (black, blue, green, and red), with rotation traces overlaid for 17 different beads. Inset: representative tracking of one microbead attached to the DNA lever. Adapted from Ref. [33] with the permission under the terms of the CC-BY license.

In nature, many microorganisms possess flagella [58] and use their wave or rotation to promote their motion. Artificial flagella synthesized from alloy [59] or glass [60] have been used to explore the mechanism of flagella in microbial movement systematically. Maier and coworkers developed a magnetically propelled bionic microswimming robot driven by a rotating external magnetic field [54]. More specifically, the bionic microswimmer consisted of a magnetic bead (diameter: 1 *μ*m) and multiple artificial flagella fabricated by tile-based DNA bundles (Figure 3(a)). The whole combination was actuated by a water-cooled three-axis Helmholtz coil, which was incorporated into the inverted fluorescence microscope. The Helmholtz coil generated a spatially homogeneous time-varying magnetic field to torque the magnetic beads (Figure 3(a) Right side). They optimized the length and number of DNA flagella bundles to improve the propulsion speed of the DNA microswimmer. In their study, the fastest bionic DNA microswimmer observed was driven at 3 Hz and propelled with a speed of 0.6 *μ*m/s. To better understand and improve the assembly process of artificial flagella, they also proposed using magnetic tweezers further to explore the real-time formation dynamics of DNA origami bundles.

The mechanical manipulating of microscale magnetic particles through an externally applied magnetic field has been extensively developed [61–63]. It is challenging to shrink the magnetic particles to the nanoscale to match whole mechanical nanodevices while generating sufficient forces to overcome thermal fluctuations [64]. To solve these problems, Lauback and coworkers innovatively assembled rigid DNA levers with a persistence length of more than 20 *μ*m to match the microscale superparamagnetic particles [33]. With the assistance of total internal reflection fluorescence (TIRF), the DNA lever system attached with fluorophores, respectively, exhibited continuous rotational motion and a specific range of angular motion. More concretely, their strategy could not only directly manipulate DNA origami nanodevices with subsecond response times but also allow precise control over the angular conformation with the resolution of ±8°, continuous rotational motion up to 2 Hz (Figure 3(b)). Furthermore, these DNA lever systems were also integrated with DNA nanorotors and nanohinges to construct the DNA rotor systems and hinge systems [33].

## 4. Biomedical Applications of Magnetic DNA Origami Carriers

**Figure 4:**
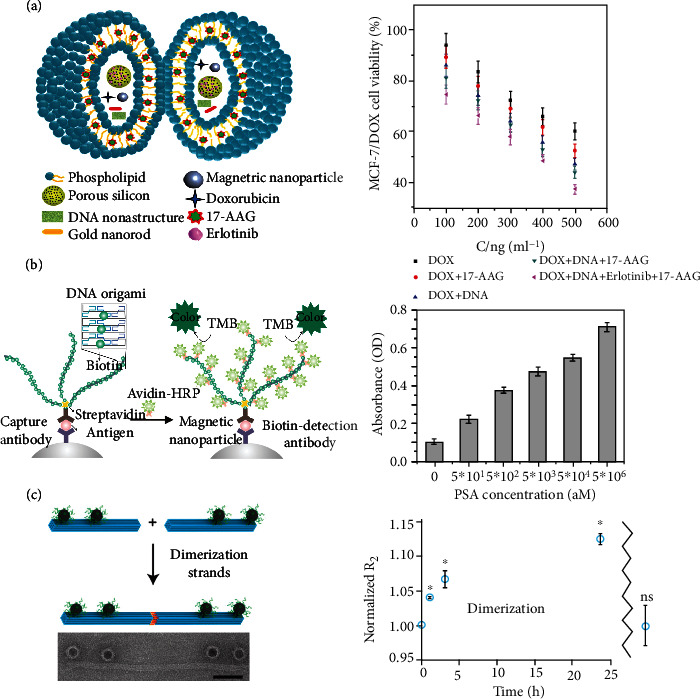
Biomedical applications of DNA origami carriers controlled by magnetic fields. (a) The multifunctional liposomes for coloading and codelivery of DNA nanostructures and drugs. Right chart: the cell viability of DOX, DOX+17-AAG, DOX+DNA, DOX+DNA+17-AAG, or DOX+DNA+Erlotinib+17-AAG was determined on DOX-resistant MCF-7/DOX cells for 24 h incubation at 37°C using a live/dead assay. Reprinted with permission from Ref. [29]. Copyright 2015 Wiley. (b) Schematic illustration of a novel signal amplification assay applying DNA belts into magnetic bead-based ELISA strategy and concentration-response for PSA detection with DNA belt-based signal amplification assay. Reprinted with permission from Ref. [32]. Copyright 2014 American Chemical Society. (c) Both schematic (top) and representative TEM image (bottom) of 16HB-IONP dimerization allow for the real-time switching of the number of IONPs/structures. Scale bar: 50 nm. Right chart: Relative changes in T_2_ relaxation rate between 16HB2(1/6)-IONP samples with and without adding dimerization strands over time, showing an increase in *R*_2_ over time for a sample containing dimerization strands. No difference (ns) between samples was observed following heat-based denaturation of 16HB support and release of free IONPs. Results were presented as the mean ± SD. Two independent experiments were analyzed. ^∗^*P* < 0.05. Adapted with permission from Ref. [77]. Copyright 2020 American Chemical Society.

The DNA origami-based platform for targeted drug delivery is one of the hottest areas in biomedicine due to its biocompatibility and programmability. To suppress the multidrug resistance of cancer cells by using the codelivery of DNA nanostructures and anticancer drugs, Kong and colleagues encapsulated nanomagnetite (around 15 nm by scanning electron microscopy (SEM); zeta-potential of +7.0 mV), hydrophilic doxorubicin (DOX), and gold nanorods (approximately 50 nm by SEM; zeta-potential of -0.6 mV) into giant liposomes (Figure 4(a)). The multifunctional liposomes could not only exhibit magnetic and thermal response characteristics but also significantly reduce the cell survival rate of DOX-resistant MCF-7/DOX cells by inhibiting multidrug resistance [29]. In addition to using DNA as the original material for assembling the nanoplatform, Guo and coauthors also succeeded in embedding magnetic nanoparticles into RNA nanoflowers by biotin-avidin conjugation [65]. To further enhance the targeting of magnetically responded RNA nanoflowers, they used folic acid to modify the RNA nanoflowers. Both the chemotherapeutic drug DOX and the photosensitizer 5,10,15,20-tetrakis(1-methylpyridinium-4-yl) porphyrin (TMPyP_4_) [66] were attached to these nanoflowers for drug delivery. Besides, they stated that the integrated system could be used as a probe to detect cancer cells with a detection limit of 50 Hela cells.

DNA origami assemblies usually employ the long single-stranded DNA derived from bacteriophage viruses [37] as the scaffold DNA. To simplify scaffold DNA design and time-consuming annealing protocols and reduce the staple strands required for synthesis [67], Beyer and coauthors designed a rolling circle amplification- (RCA-) based ssDNA [68, 69] containing hundreds of short periodic sequences that can be easily prepared through the isothermal and highly efficient RCA reaction [32]. Using this ssDNA, they synthesized DNA belts. They combined DNA belts with magnetic bead-based enzyme-linked immunosorbent assay (ELISA) (Figure 4(b)) for the high-sensitivity detection of PSA [70], a serum biomarker available for screening prostate cancer [71]. They argued that the detection limit of this signal amplification mechanism for PSA was 50 aM (Figure 4(b)). Furthermore, with the help of streptavidin magnetic beads and specific aptamer [72, 73] targeting PSA, Wei and colleagues developed a DNA triangular prism for ultra-sensitive detection of PSA. The presence of PSA induced the assembly of DNA origami, resulting in strong fluorescence. Without PSA, DNA triangular prism cannot be formed, thereby reducing the fluorescence intensity. In their research, the proposed DNA triangular prism possessed a wide linear range of 200-300 pg/ml, and the detection limit was 30 pg/ml [27].

Up to now, magnetic resonance imaging (MRI) has become one of the most effective diagnostic strategies for inspecting lesions in clinical applications. MRI contrast agents enable primitive MRI machines to distinguish tumor specificity. Due to biocompatibility and unique magnetic properties [74], magnetic iron oxide nanoparticles (IONPs) have been developed as MRI contrast agents and magnetically guided carriers for drug and gene delivery. To improve the activity of IONPs as MRI contrast agents, the most conventional assembly method was to gather them together and significantly increase the T_2_ relaxivity of the particles [75, 76]. Meyer and coauthors employed a DNA origami rod composed of 16HB for constructing anisotropic assemblies of IONPs with a high degree of control and precision over the spatial organization of the particles [77]. They realized the cluster modularization of IONPs and then adjusted the MRI contrast generation efficiency by changing the number and spacing of IONPs. They also innovatively synthesized a DNA origami dimer (Figure 4(c)) and found that the dynamic assembly of DNA origami dimers could drive the dynamic changes of T_2_ relaxivity (Figure 4(c), right chart). Moreover, Rafati and coworkers utilized a DNA nanotube to array magnetic nanoparticles, which are precisely placed in predetermined positions at the exterior surface of the DNA nanotube [78].

## 5. Electromagnetic Applications of Gold Nanoparticles Arrayed by DNA Platforms

**Figure 5:**
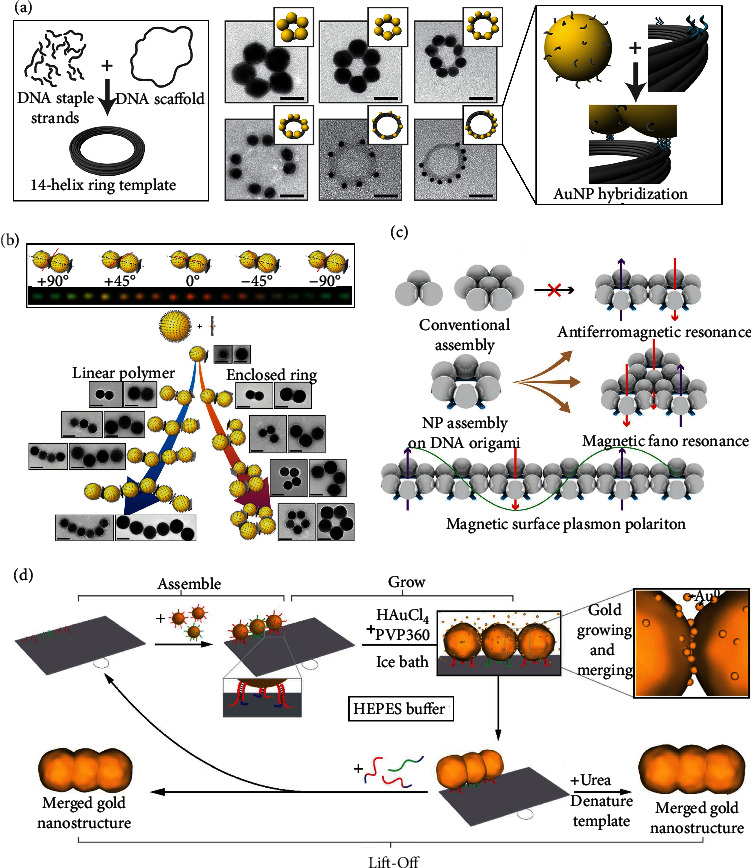
Regular arraying and assembly of gold nanoparticles using DNA origami. (a) Left diagram: self-assembly of DNA origami nanoparticle rings. Right graph: schematic illustration and TEM images of 14HB with multiple AuNPs. Enlarged panel: DNA-functionalized AuNPs were attached to specified positions on the 14HB via hybridization of the handle sequences protruding from the 14HB and the complementary DNA strands on the AuNPs. Scale bars: 40 nm. Adapted with permission from Ref. [34]. Copyright 2015 American Chemical Society. (b) Top panel: dark-field images concerning the angular deviation between the incident electric field (red axis) and long-axis of the dimer. Bottom panel: route for metamolecular assembly of 3D DNA origami and AuNSs (60–100 nm) together with representative TEM images of each assembled motif. Scale bars: 100 nm. Reprinted with permission from Ref. [80]. Copyright 2018 Wiley. (c) Programmable assembly of DNA origami leads to complex magnetic plasmon architectures, including magnetic surface plasmon polariton. Adapted with permission from Ref. [81]. Copyright 2019 Wiley. (d) Schematic diagram of employing the Assemble, Grow, and Lift-Off (AGLO) strategy to construct a predesigned gold nanostructure. Reprinted with permission from Ref. [82]. Copyright 2020 Royal Society of Chemistry.

Many publications [34, 79–82] showed the usage of the DNA origami platform to arrange the gold nanoparticle (AuNP) into the specific conformation, which could generate artificial magnetism and affect the inherent optical properties. AuNP is one of the most critical nanomaterials in the biomedical fields due to low cytotoxicity, high biological stability, and unique optical properties [83]. Concretely, AuNPs show colorimetric changes varying from the dispersed state to the aggregated state under the influence of localized surface plasmon resonance [84]. To construct a ring resonator [85] composed of the AuNPs that can exhibit electric and magnetic resonance, Roller and colleagues skillfully applied DNA origami technology to synthesize the ring resonator skeleton [34]. They used software caDNAno [86] to design DNA bundles with 14 parallel arranged DNA double helices of 200 nm length and then bend the bundles into DNA nanoring by deleting or adding bases at specific sites (Figure 5(a)). In their study, varying amounts of AuNPs (40 nm, 30 nm, and 20 nm) were successfully hybridized [87, 88] on the DNA nanoring (Figure 5(a)). Both scattering spectroscopy and computational simulations demonstrated that the electrical and magnetic resonance responses of different plasmonic rings were susceptible to their external geometric configurations. More importantly, visible frequencies might support these resonances of ring resonators, including dipolar, multipolar, and magnetic modes.

To figure out the limitation of self-assembled plasmonic metamaterials caused by the nonuniformity of large gold nanospheres (AuNSs) [34, 89], Lee and colleagues successfully produced the roundest, highly uniform, and smooth AuNSs with the size of 60-100 nm, which tolerated high-salt conditions (i.e., 20∗10^−3^ M Mg^2+^) after attaching oligonucleotides to their surfaces [80]. Using DNA origami-enabled assembly, AuNSs (60 nm) were hybridized with the top and bottom sides of a nanopegboard to form plasmonic dimers. Interestingly, according to the dark-field optical microscope images of the plasmonic dimers obtained by the dynamic angle between the incident electric field and long-axis, a gradual change from greenish to reddish scattering colors via rotation of the dimer was observed, which was consistent with theoretical predictions (Figure 5(b), top panel). They also fabricated symmetric tetramer ring and linear oligomeric chains and characterized their plasmonic resonance behaviors through dark-field scattering and theoretical calculations (Figure 5(b)). Due to the high fidelity of the overall assemblies, the plasmonic assemblies generated strong electric and unnatural magnetic resonances. Furthermore, they also designed a DNA origami hashtag tile that polymerizes into rigid one-dimensional chains. They further explored that the plasma polymers based on these DNA origami chains effectively transported plasma angular momentum and magnetic surface plasmonic polaritons at the deep-subwavelength scale [79].

Magnetic field manipulation at the nanoscale has always been a pursuit of plasmonic metamaterials, as it can achieve attractive optical properties such as high-sensitivity circular dichroism [90], directional scattering [91], and low refractive index [92]. Inspired by the natural magnetism of cyclic aromatic molecules, the regular ring clusters of plasmonic nanoparticles might be a promising assembly for inducing artificial magnetism. Wang and colleagues used a DNA origami hexagon tile [93] to assemble six AuNPs into a monocyclic ring structure [81]. They skillfully deposited the silver onto a flat substrate to further expand the volume of nanoparticles and ensure the integrity of the entire system [94]. The emergent properties of magnetic plasmons could also be realized, such as antiferromagnetism, a purely magnetic Fano resonance, and magnetic surface plasmon polaritons (Figure 5(c)). In addition to depositing silver onto AuNPs [81], Luo and coworkers chose to deposit gold onto the surface of AuNPs, which were conjugated with a simple 2D DNA origami sheet [82]. After slow and continuous deposition, these AuNPs merged with adjacent particles to form a constant gold nanostructure in a predesigned shape. 80% gold nanostructures were eventually lifted off the origami template. Hence, they called these innovative methods: Assemble, Grow and Lift-Off strategy (Figure 5(d)). Moreover, the excess AuNPs and DNA components could be easily recovered with the coordination of the magnetic beads.

## 6. Conclusions

Up to now, the rapid progress of micro/nanomanipulation technology, bionanomaterials, and robotics have facilitated the emergence of the interdisciplinary field of micro/nanorobotics [95–97]. Magnetic micro/nanorobots have obtained increasingly complex motion trajectories under the control of changing magnetic fields. These micro/nanoscale motions have been applied to targeted transportation, minimally invasive surgery, and intelligent induction [98, 99]. The highly programmable DNA origami allows us to assemble DNA frameworks with any shape at the micro/nanometer level. Recently, the number of scientific researches on DNA origami engineered micro/nanorobots in response to magnetic controls has shown a rapid growth trend. Among these researches, covalent modified magnetic beads dominated the sensing modules of almost most magnetically actuated DNA micro/nanorobots. Some notable original works [33, 54] realized the controlled motions and rotations of specific micro/nanorobots with the magnetic beads help. Apart from mechanical movements, numerous novel applications on magnetic DNA micro/nanorobots in biosensing and drug delivery were discovered [27, 29, 32, 65]. Driven by the external magnetic field, some studies [31, 43, 48] indicated the high throughput purification for targeted objects. Several papers [28, 30, 55] demonstrated that the magnetic beads-loaded DNA origami structures could characterize and measure the bend or twist forces of whole systems on a piconewton scale. To impact the magnetic properties of DNA micro/nanorobots innovatively, Opherden and colleagues successfully decorated DNA triangle and six-helix buddle with Eu^3+^ ions to provide an effective tool for magnetic manipulation of DNA nanodevices without relying on any covalent modifications [100].

Along with the development and cooperation of multidisciplinary technology, the synthesis of magnetic micro/nanorobots will develop in a low-cost and straightforward direction. Previously, low cost was a significant obstacle to the assembly of nanodevices in DNA origami engineering. Fortunately, the successful production of ssDNA based on RCA has resolved this problem to a certain extent [32]. The biocompatibility and ethical safety of magnetic DNA micro/nanorobots are also crucial elements that must be considered. Thus, most current research is in its infancy in practical applications. We believe that with the continuous optimization of DNA micro/nanorobot assembly solutions and the constant miniaturization and intelligentization of magnetic field systems, more magnetically driven DNA micro/nanorobots will eventually be used *in vitro* studies and clinical applications.

## Data Availability

The original data supporting this review are from previously reported studies and datasets, which have been cited. The processed data are available from the corresponding author upon request.
